# Hydrogen sulfide production during early yeast fermentation correlates with volatile sulfur compound biogenesis but not thiol release

**DOI:** 10.1093/femsyr/foad031

**Published:** 2023-06-05

**Authors:** Ruoyu Hou, Rebecca E Jelley, Katryna A van Leeuwen, Farhana R Pinu, Bruno Fedrizzi, Rebecca C Deed

**Affiliations:** School of Chemical Sciences, University of Auckland, 23 Symonds St, Auckland 1010, New Zealand; School of Biological Sciences, University of Auckland, 3A Symonds Street, Auckland 1010, New Zealand; School of Chemical Sciences, University of Auckland, 23 Symonds St, Auckland 1010, New Zealand; School of Chemical Sciences, University of Auckland, 23 Symonds St, Auckland 1010, New Zealand; Biological Chemistry & Bioactives, The New Zealand Institute for Plant and Food Research Limited, Private Bag 92169, Auckland 1142, New Zealand; School of Chemical Sciences, University of Auckland, 23 Symonds St, Auckland 1010, New Zealand; School of Chemical Sciences, University of Auckland, 23 Symonds St, Auckland 1010, New Zealand; School of Biological Sciences, University of Auckland, 3A Symonds Street, Auckland 1010, New Zealand

**Keywords:** Fermentation, hydrogen sulfide, *Saccharomyces cerevisiae*, 3-sulfanylhexan-1-ol, volatile sulfur compounds

## Abstract

Yeasts undergo intensive metabolic changes during the early stages of fermentation. Previous reports suggest the early production of hydrogen sulfide (H_2_S) is associated with the release of a range of volatile sulfur compounds (VSCs), as well as the production of varietal thiol compounds 3-sulfanylhexan-1-ol (3SH) and 3-sulfanylhexyl acetate (3SHA) from six-carbon precursors, including (*E*)-hex-2-enal. In this study, we investigated the early H_2_S potential, VSCs/thiol output, and precursor metabolism of 11 commonly used laboratory and commercial *Saccharomyces cerevisiae* strains in chemically defined synthetic grape medium (SGM) within 12 h after inoculation. Considerable variability in early H_2_S potential was observed among the strains surveyed. Chemical profiling suggested that early H_2_S production correlates with the production of dimethyl disulfide, 2-mercaptoethanol, and diethyl sulfide, but not with 3SH or 3SHA. All strains were capable of metabolizing (*E*)-hex-2-enal, while the F15 strain showed significantly higher residue at 12 h. Early production of 3SH, but not 3SHA, can be detected in the presence of exogenous (*E*)-hex-2-enal and H_2_S. Therefore, the natural variability of early yeast H_2_S production contributes to the early output of selected VSCs, but the threshold of which is likely not high enough to contribute substantially to free varietal thiols in SGM.

## Teaser

Endogenous H2S produced during early fermentation by yeast correlates with volatile sulfur compound formation; however, these H2S concentrations are insufficient to result in varietal thiol production via the (E)-hex-2-enal pathway.

## Introduction

Hydrogen sulfide (H_2_S) produced by yeast has been suggested to play important roles in heavy metal detoxification (Kikuchi 1965, Ono et al. 1991), population synchrony (Sohn et al. 2000, Kwak et al. 2003), and chronological longevity (Hine et al. 2015). Yeast-derived H_2_S is mainly produced from exogenous sulfate via the sulfur assimilation pathway (SAP) in response to the metabolic requirement for sulfur-containing amino acids (Linderholm et al. 2008, Rose et al. 2017), cysteine catabolism (Stipanuk 2004), and from the application of elemental sulfur under an anaerobic and low-pH environment (Araujo et al. 2017). Of note, H_2_S has been suggested to play metabolic and protective roles in the cell during the early phase of fermentative transition, where a time-critical nutritional switch and oxidative stress response occur (Jiranek et al. 1995, Kwak et al. 2003).

While commonly considered to be an undesirable off-odor in wines, early production of H_2_S has been linked to the accumulation of thiol compounds, specifically 3-sulfanylhexan-1-ol (3SH) and its acetate ester derivative, 3-sulfanylhexyl acetate (3SHA), in the presence of plant-derived α,β-unsaturated aldehydes and alcohols, via direct addition to the double bond (Schneider et al. 2006, Harsch et al. 2013). However, the contribution of this pathway to total thiol output is disputed (Schneider et al. 2006, Subileau et al. 2008, Roland et al. 2010, Harsch et al. 2013, Bonnaffoux et al. 2018). Interestingly, the concentrations of grape-derived α,β-unsaturated six-carbon precursors, such as (*E*)-hex-2-enal and its derivatives, rapidly diminish within a few hours of yeast inoculation (Harsch et al. 2013), potentially due to active yeast detoxification (Trotter et al. 2006). This process likely occurs before inoculation in naturally harvested and pressed grape must, due to interactions with indigenous communal microorganisms (Joslin and Ough 1978, Hammerbacher et al. 2019). Alternatively, free (*E*)-hex-2-enal, like other α,β-unsaturated aldehydes, can be scavenged by plant-derived glutathione S-transferase, or detoxified by plant-derived enzymes including aldehyde dehydrogenase, aldo-keto reductase, NADPH-dependent 2-alkenal reductase, and alkenal/one oxidoreductase, albeit in a relatively minor and slower process (Mano et al. 2002, 2019, Yamauchi et al. 2011, Mano 2012). The window of precursor-H_2_S concurrence is, therefore, considered limited to the immediate-early phase of the fermentation (<12 h), when H_2_S production begins to increase. Moreover, we previously observed the accumulation of a range of volatile sulfur compounds (VSCs), concurrent with H_2_S production, during the course of laboratory fermentation in synthetic grape medium (SGM) (Kinzurik et al. 2016). Specifically, chemical profiling of finished ferments produced from full-length fermentations revealed that the levels of ethanethiol, *S*-ethyl acetate, and diethyl disulfide in the final wine were associated with H_2_S production. Tracing of isotope incorporation further suggested that these compounds are direct downstream products of H_2_S (Kinzurik et al. 2016). Moreover, exogenous H_2_S spiking into actively fermenting yeast suggested yeast-mediated production of specific downstream VSCs (Kinzurik et al. 2020).

The above findings highlight the need to further understand the role of H_2_S during the early stage of yeast fermentation, where a dynamic physiological transition takes place inside yeast cells. Indeed, it is well documented in previous reports that remarkable inter-strain variabilities in H_2_S potential are observed among *Saccharomyces cerevisiae* yeasts, including natural isolates, commercial strains, and laboratory deletants (Kumar et al. 2010, Winter et al. 2014). However, such reports are usually insufficient in terms of displaying detailed temporal resolution of H_2_S biogenesis, nor do they attempt to associate H_2_S evaluation with quantitative chemical profiling during fermentation. Although we have qualitatively established the association between H_2_S with its immediate downstream products (Kinzurik et al. 2015), this previous work did not differentiate the impact of H_2_S on VSC production at the early stage of fermentation from finished wine samples due to the technical limitations of silver nitrate tester tubes. Given this approach directly quantifies the liberated H_2_S pool, it therefore can only detect and quantify H_2_S after ∼18 h of fermentation.

In this work, we aimed to couple the early stage (12 h) H_2_S profiles of a panel of laboratory reference and commonly used commercial wine yeast strains, measured via methylene blue reduction assay, alongside VSC and volatile thiol quantification in model juice using gas chromatography-mass spectrometry (GC-MS) and liquid chromatography-mass spectrometry (LC-MS), in an attempt to characterize the early H_2_S-VSC-thiol profile. Furthermore, the relationships among early H_2_S production, downstream VSC formation, and final thiol output contributed from a C_6_ precursor, specifically (*E*)-hex-2-enal, were assessed for potential applications in yeast phenotype screening for the wine industry.

## Materials and methods

### Yeast strains

Two laboratory reference strains, BY4743 (*MATa/α his3Δ1/his3Δ1 leu2Δ0/leu2Δ0 LYS2/lys2Δ0 met15Δ0/MET15 ura3Δ0/ura3Δ0*) and BY4741 (*MATa his3Δ1 leu2Δ0 met15Δ0 ura3Δ0*) (EUROSCARF, Germany), along with nine commercially available wine strains or vineyard isolates: Lalvin EC-1118 (Lallemand, Canada), Zymaflore F15 (Laffort, France), Enoferm M2 (Scott Laboratories, USA), Maurivin MaxiThiol (AB Biotek, New Zealand), RM11 (Princeton University, USA), Maurivin UCD522 (AB Biotek, New Zealand), Anchor VIN13 (Scott Laboratories, USA), Zymaflore VL3 (Laffort, France), and Zymaflore X5 (Laffort, France), were included in this work. For early precursor kinetics, BY4741 and the BY4741-*Δoye2 Δoye3* double deletant were used (EUROSCARF, Germany). Before each experiment, individual colonies from streaked plates of cryopreserved stocks were inoculated in liquid YPD medium (10 g L^−1^ yeast extract, 20 g L^−1^ casein peptone, and 20 g L^−1^ D-glucose) using standard microbiological procedures, and incubated overnight at 28 °C, with adequate aeration and orbital shaking at 120 rpm.

### Media and reagents

All chemicals, unless otherwise indicated, were purchased from Sigma-Aldrich (Germany). Analytical-grade anhydrous ethanol was purchased from Ajax Finechem (Taren Point, NSW, Australia), and D-glucose was purchased from Merck (Kenilworth, NJ, USA). The internal standard dimethyl-*d*_6_ sulfide (*d*_6_-DMS), along with methionol, methanethiol, and dimethyl disulfide, were purchased from Sigma-Aldrich (Darmstadt, Germany). Liquid YPD or YPD agar plates were used for routine maintenance of standard yeast cultures, while chemically defined SGM mimicking grape juice (21°Brix, pH 3.2, yeast assimilable nitrogen 300 mg L^−1^) was used for fermentative growth, as described in previous reports (Kinzurik et al. 2015, Deed et al. 2019). To cater for the methionine auxotrophy of the BY4741 strain, 10-fold concentrations of L-methionine were supplemented in the final SGM (0.3 mM). (*E*)-hex-2-enal (Sigma-Aldrich, Darmstadt, Germany) stock solution was freshly prepared and aliquoted by dissolving in anhydrous ethanol at 1.5 mg mL^−1^ and supplemented into the medium preparation at a final concentration of 1.5 mg L^−1^ just before yeast seeding. YNB medium (yeast nitrogen base without amino acids or ammonium sulfate 1.7 g L^−1^, ammonium chloride 0.3 g L^−1^, L-malic acid 3 g L^−1^, citric acid 0.2 g L^−1^, sucrose 200 g L^−1^, pH = 3.2) was used for early precursor kinetics.

### Methylene blue reduction based H_2_S detection

Methylene blue-based (MetB) H_2_S detection in live yeast cultures was performed according to previous reports (Winter and Curtin 2012, Winter et al. 2014) with minor modifications. Briefly, stationary-phase yeast precultures were prepared with overnight incubation at 28 °C in liquid YPD. In each well of a 96-well microtiter plate (Corning, USA), a 170 µl aliquot of SGM, 20 µl aliquot of a MetB reaction mix (0.5 mg mL^−1^ MetB, 50 mM citric acid buffer at pH 4.5), and 10 µl of yeast culture were mixed thoroughly to a final cell count of 2 × 10^6^ cell mL^−1^. Assays were carried out in quadruplicate with uninoculated SGM as the blank control. The microtiter plate was placed in a SpectraMax iD3 plate reader (Molecular Devices, CA, USA) with intermittent shaking (10 s at intermediate intensity for every 10 min) at 25 °C. Absorbances at 663 and 600 nm were recorded automatically at 10-min intervals for 12 h. Cell growth controls were included by recording the optical densities at 600 nm concurrently for cells without the MetB detection mix.

### Laboratory fermentation

Laboratory-scale fermentation was performed as described previously (Deed et al. 2019). Briefly, precultures of each yeast strain were prepared overnight. Yeast cells were collected by centrifugation at 3000* g* for 5 min, then washed with sterile water. Starter cultures were seeded at 2 × 10^6^ cells mL^−1^ in triplicate into 250 ml Erlenmeyer flasks sealed with water-filled airlocks, each containing 100 ml of SGM and supplemented with 1.5 mg L^−1^ of (*E*)-hex-2-enal. After 12 h of incubation at 25 °C with 120 rpm agitation, fermentations were harvested by centrifugation at 3000   *g* for 15 min. Cell-free supernatants, representing the finished wines, were collected and stored in polypropylene 70-ml plastic containers at −80 °C prior to chemical profiling.

For the early precursor kinetics experiment, overnight precultures of BY4741 and BY4741-*Δoye2 Δoye3* inoculants were seeded into YNB medium spiked with 1.5 mg L^−1^ of (*E*)-hex-2-enal at 2 × 10^6^ cells mL^−1^ final cell density. NaSH was supplemented just before yeast inoculation at a final concentration of 10 mg L^−1^ as the exogenous H_2_S donor. Samples were harvested at 1, 2, 3, 6, and 24 h of fermentation by centrifugation at 3000 *g* for 15 min. Two initial samples for each fermentation run were also collected before (0 h medium only) and immediately after yeast inoculation (0 h).

### VSC quantification via HS-SPME/GC-MS

VSCs in finished wines were extracted using the method developed and employed in previous reports (Nguyen et al. 2012, Kinzurik et al. 2015, Deed et al. 2019). Defrosted wine sample (10 ml) was saturated with magnesium sulfate heptahydrate (2.6 g) and purged with nitrogen to prevent analyte degradation, followed by injection of 50 µl of an internal standard mix (30 µg L^−1^*d*_6_-DMS, 2 µg L^−1^ DPDS, 547 µg L^−1^ 3-methytlthio-1-hexanol). Head space solid phase micro-extraction coupled with gas chromatography-mass spectrometry (HS-SPME/GC-MS) on an Agilent Technologies 7890 GC system coupled with a 5975C inert XL MSD (Agilent, Santa Clara, CA, USA) was used for quantitation of the internal standards and the VSCs. The SPME fiber was composed of divinylbenzene/carboxen-polydimethylsiloxane (DVB/CAR-PDMS, 50/30 μm × 2 cm) (Supelco Bellefonte, PA, USA). The separating column included a 30 m × 0.320 mm × 0.25 μm HP-1MS coupled with a 30 m × 0.320 mm × 0.25 μm HP-Innowax fused silica capillary column (Agilent, J&W Scientific, New Zealand). Pre-sampling agitation of GC vials was provided using a Gerstel agitator/stirrer controlled by MAESTRO software (version 1.2.0) (Gerstel, Mülheim an der Ruhr, Germany). The MassHunter Workstation software (version B.07.01) (Agilent, Santa Clara, CA, USA) was used to identify peak areas corresponding to the signature ions for each compound based on previous reports and the NIST library reference (Nguyen et al. 2012, Kinzurik et al. 2015). Concentrations were determined from a calibration curve prepared in model wine (12% v/v ethanol, 5 g L^−1^ L-tartaric acid, pH 3.2), spiked with gradient concentrations of the compounds analyzed.

### Thiol quantification via SPE/GC-MS and QuEChERS-based extraction followed by LC-MS/MS

Thiol compound quantification was carried out using ethyl propiolate (ETP) derivatization followed by C-18 solid phase extraction (SPE) and subsequent GC-MS analysis of enriched samples, as outlined in our previous work (Herbst-Johnstone et al. 2013). Calibration curves for 3SH and 3SHA were prepared over the ranges of 0–27 500 ng L^−1^ for 3SH and 0–3400 ng L^−1^ for 3SHA (*R*^2^ > 0.995). MassHunter Workstation software (version B.07.01) (Agilent, Santa Clara, CA, USA) was used for data integration.

Thiol quantification was also performed using the QuEChERS-based extraction followed by liquid chromatography-mass spectrometry (LC-MS/MS), as outlined in our previous works (Tonidandel et al. 2021, Jelley et al. 2022).

### Data analysis

The cumulative H_2_S profile of each strain was estimated as described in previous reports (Winter and Curtin 2012). GC-MS sample analysis was performed using the MassHunter workstation software (version B.07.01, Agilent, Santa Clara, CA, USA). Correlations between H_2_S production and VSC production from the early fermentation period were tested using Spearman’s rank test (*P* < .05) and the 95% CI estimated using jackknife Euclidean likelihood-based inference (de Carvalho and Marques 2012). One-way ANOVA with post hoc Tukey’s HSD test (*P* < .05) was performed to analyze strain variability in precursor metabolism.

## Results and discussion

This work evaluates the production of H_2_S during the early stage of alcoholic fermentation using a collection of laboratory reference and commercial yeast strains fed with chemically defined SGM (Deed et al. 2019). Previous studies have shown that early H_2_S release in the presence of the short-lived unsaturated C_6_ compound (*E*)-hex-2-enal induces direct, yeast-mediated, formation of varietal thiols, including 3SH/3SHA (Harsch et al. 2013). It is therefore interesting to evaluate whether variation in *S. cerevisiae* early H_2_S-producing potential is associated with thiol-producing phenotypes in the presence of (*E*)-hex-2-enal.

### Early H_2_S profiles during fermentation of the laboratory and commercial strains

**Figure 1.**
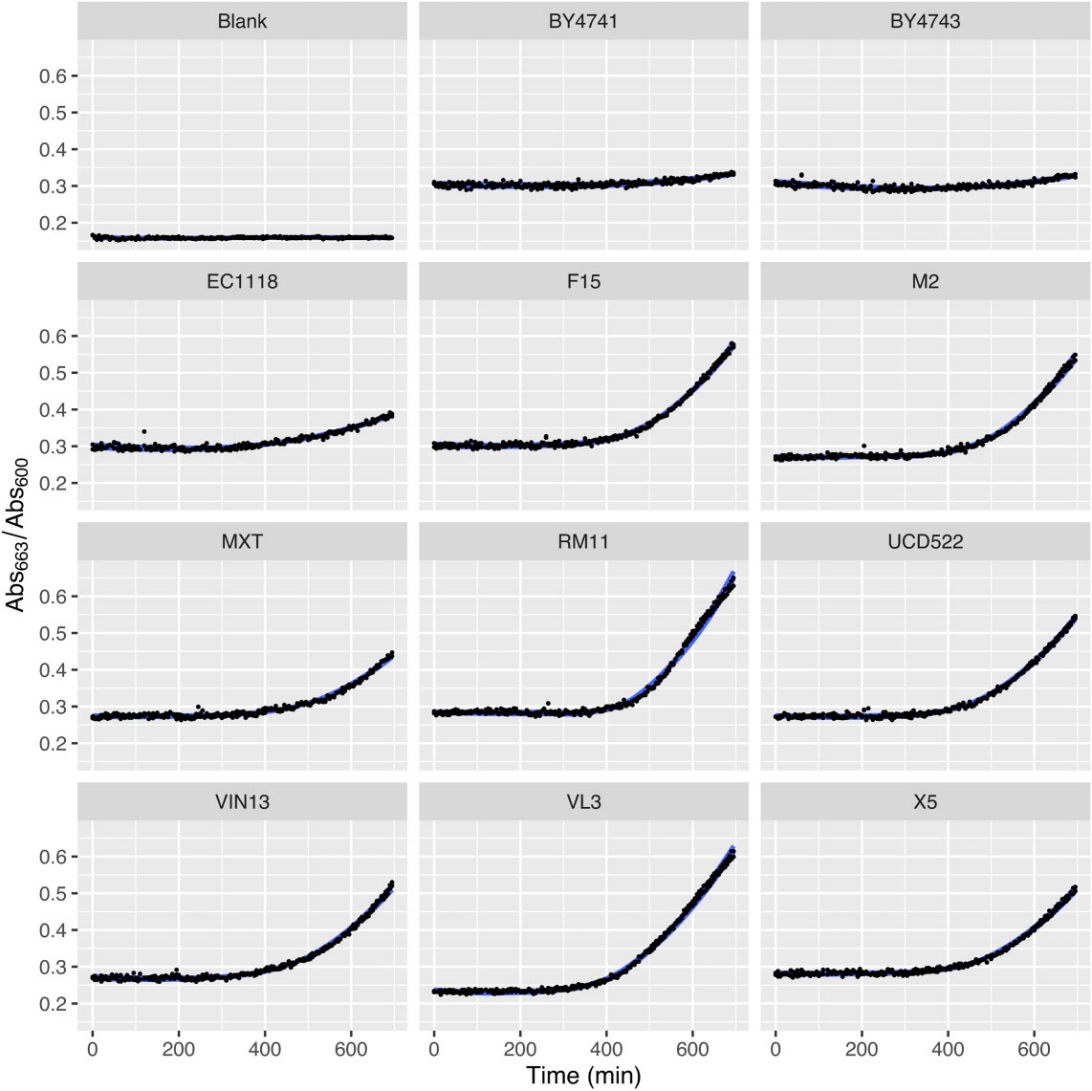
Mean H_2_S accumulation of 11 laboratory reference and commercial *S. cerevisiae* strains in SGM during the initial 12 h of fermentation (*n* = 5). Plot shows 12 h time-course measurements of Abs_663_/Abs_600_ indicating MetB clearance normalized with biomass growth at 10 min intervals. Strain names are denoted at the top of each plot.

We surveyed the early H_2_S production profile of 11 laboratory reference and commonly used commercial yeast strains (two laboratory reference strains: BY4741 and BY4741, and nine commercial strains: EC1118, F15, M2, MaxiThiol, RM11, UCD522, VIN13, VL3, and X5). Significant variability in the H_2_S-producing potential of the 11 selected strains was demonstrated as early as 12 h after initiation of fermentation (Fig. 1) using time-course spectrophotometry with the MetB reduction method. H_2_S released from the ferments was below the detection threshold of conventional H_2_S detector tubes (Kinzurik et al. 2015, Huang et al. 2016). Laboratory reference strains showed lower H_2_S accumulation compared with the commercial strains. Of note, BY4741 has previously been reported to be a high producer of H_2_S in terms of total H_2_S quantification over the course of a full-length fermentation (Kinzurik et al. 2015) compared with its BY4743 diploid counterpart. It is hypothesized that the low accumulation of H_2_S in BY4741 fermentations observed in the current study, and generally in fermentations produced by the laboratory reference strains, arises from lower adaptiveness to the fermentative environment (high sugar, low pH) (Spiropoulos et al. 2000, Linderholm et al. 2008). Consistent measurements were obtained for previously reported moderate-high H_2_S producers, including F15 and UCD522 (Mendes-Ferreira et al. 2010, Kinzurik et al. 2016, Xing and Edwards 2019). H_2_S quantifications in previous reports have shown extensive variation in H_2_S production among a vineyard yeast collection (Spiropoulos et al. 2000, Mendes-Ferreira et al. 2002), as well as among single gene deletants (Winter et al. 2014). In these studies, most of which aimed at identifying low-H_2_S-producing strains that are favorable in winemaking practice, H_2_S profiles are usually reported with less temporal resolution as an accumulation within at least 2–3 days. In contrast, our data addresses the H_2_S profile at the early transitional phase of fermentation with a snapshot suggesting discernible variabilities before stationary fermentation is plateaued.

### Correlation of early H_2_S, VSCs, and thiol production

**Figure 2.**
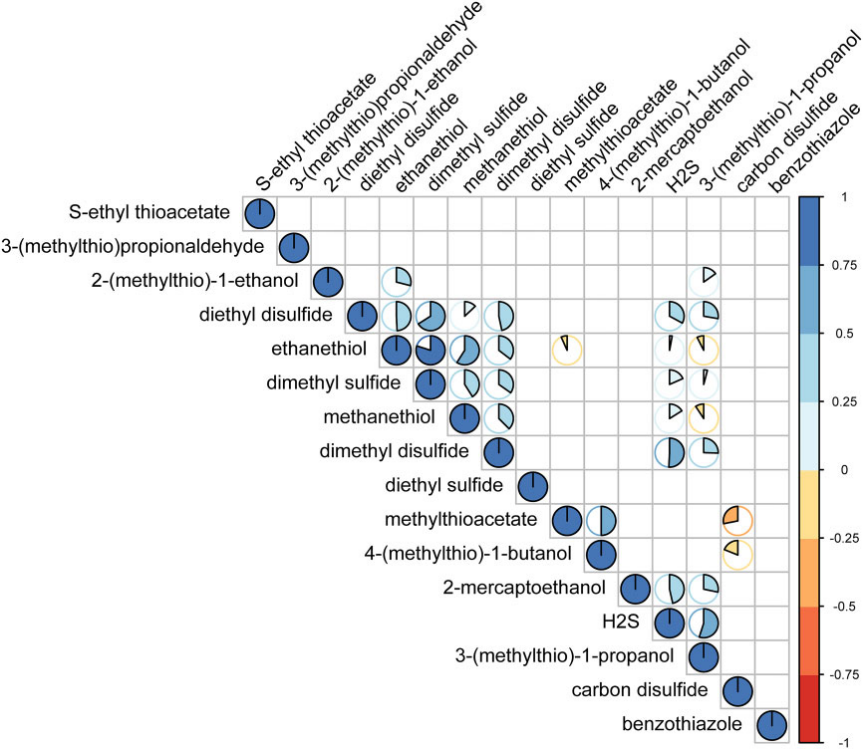
Correlation of H_2_S and VSC production during early fermentation in SGM (*n* = 3). Concentrations of dimethyl disulfide [*ρ* = 0.508, 95% CI (0.184, 0.830)], 2-mercaptoethanol [*ρ* = 0.465, 95% CI (0.142, 0.785)], and diethyl disulfide [*ρ* = 0.329, 95% CI (0.003, 0.0.652)] in 12 h ferments were positively correlated to the H_2_S profile, respectively (Spearman’s rank correlation, *P* < .05).

The concentrations of VSCs for each strain produced at 12 h fermentation were profiled and coupled with the 12 h H_2_S production measurement (Fig. 2). For all yeast strains tested, the early accumulation of dimethyl disulfide [DMDS, *ρ* = 0.508, 95% CI (0.184, 0.830)], 2-mercaptoethanol [2-ME, *ρ* = 0.465, 95% CI (0.142, 0.785)], and diethyl sulfide [DES, *ρ* = 0.329, 95% CI (0.003,0.0.652)], were positively correlated with H_2_S production (Fig. 2).

The accumulation of several VSCs, including ethanethiol (EtSH), S-ethyl thioacetate (ETA), and diethyl disulfide (DEDS), during a full-length fermentation has previously been reported to be the result of H_2_S production (Kinzurik et al. 2016). ^34^S isotope labeling provided evidence of a direct association between H_2_S and these VSCs in the finished wine. H_2_S is thought to react with either acetaldehyde or ethanol to produce EtSH (Rauhut and Kurbel 1994), which then dimerizes to produce DEDS in a yeast-independent manner (Bobet et al. 1990), as is consistent in this experiment. DMDS is suggested to be a downstream derivative of methanethiol (MeSH). While MeSH itself was observed in the current study to be only marginally correlated with H_2_S production [*ρ* = 0.161, 95% CI (−0.245, 0.566)], MeSH is the direct product of methionine catabolism (Landaud et al. 2008, Deed et al. 2019), and is spontaneously oxidized/dimerized into DMDS (Chin and Lindsay 1994, Kreitman et al. 2017). Notably, exogenous H_2_S spiking into yeast-containing active fermentation leads to increased MeSH (Kinzurik et al. 2020). This can be interpreted as an indirect result from increased methionine availability via yeast SAP, thus associated with elevated MeSH production via demethiolation in the feedback regulation (Arfi et al. 2002, Perpète et al. 2006), which further increase the concentration of DMDS, its immediate downstream oxidant.

The association of ETA with H_2_S production during fermentation was previously suggested (Kinzurik et al. 2016), whereas a recent study reveals that ETA rapidly decreases in the presence of yeast cells (Jiménez-Lorenzo et al. 2022). Our data, however, could not establish a positive correlation between H_2_S and ETA. In contrast, the correlation of H_2_S with DEDS suggests the dominance of the alternative pathway of the fate of EtSH. Interestingly, there is evidence of the reverse conversion of DEDS to EtSH (Bobet et al. 1990), ETA to EtSH, and DMDS to MeSH occurring in post-bottling finished wine (Bekker et al. 2018).

2-ME is proposed to be the production of cysteine Ehrlich degradation (Silva Ferreira et al. 2003, Vermeulen et al. 2006). Although a range of reports has accounted for its quantification in distinct wine varieties and beer (Rapp et al. 1985, Vermeulen et al. 2006, Fedrizzi et al. 2007, Jiménez-Lorenzo et al. 2022), the association of 2-ME and early H_2_S is documented for the first time. The early formation of 2-ME, much like DMDS, could be explained by the vigorous yeast metabolism during the immediate-early stage of fermentation, which may exhibit distinct profiles in finished or aging wines, as the decrease of 2-ME concentration over time invariably shown in previous investigations (Silva Ferreira et al. 2003, Fedrizzi et al. 2007).

Together, these results suggest that the concentration of downstream products correlates with the efflux of H_2_S at the very early stage of fermentation, some of which could serve as surrogate indicators when direct measurement of H_2_S is not possible. Overall, a distinct volatile metabolite landscape and pathway preference were revealed as compared with stationary phase fermentation or further process. Further longitudinal investigation is required along the course of full-scale fermentation to illustrate the dynamic interconversion of volatile compounds via distinct biological/chemical processes.

Although the SGM was spiked with (*E)*-hex-2-enal prior to fermentation, no quantifiable 3SH or 3SHA was observed in any of the 12 h fermentation samples. We cannot determine whether the absence of these thiols is attributable to the insufficient H_2_S production in the SGM during early fermentation, possibly due to the composition of the SGM, and/or due to thiol production being below the detection limits of the two methods employed. There have been limited attempts to quantify thiol production using a chemically defined SGM, and none of them, to our knowledge, have investigated the very early phase of fermentation. Santiago and Gardner quantified thiols in end-point ferments produced from SGM spiked with cysteinylated and glutathionylated precursors, and reported a maximum of 8%–10% of total thiol yield (Santiago and Gardner 2015). Jelley et al. quantified endpoint 3SH produced from SGM supplemented with grape marc extract as sources of thiol precursors at 216.2 or 1244.4 ng L^−1^ according to the levels of grape marc extract input (Jelley et al. 2020). On the other hand, there have been even fewer attempts to study the time-course evolution of 3SH/3SHA during fermentation, especially in the early phase. Tominaga et al. reported the production of these thiols from the VL3c yeast strain in Sauvignon blanc grape must over a 5 d fermentation, with the first observation (∼550 ng L^−1^ of 3SH) reported on day 2 after initiation (Tominaga et al. 1998). Bonnaffoux et al. also using Sauvignon blanc grape must, reported ∼117.5 ng L^−1^ of 3SH at 15 h after initiation (Bonnaffoux et al. 2018). In current work, two distinct quantification approaches to measure thiols were utilized. A QuEChERS-based extraction yielded very poor recovery of the organic fraction, possibly due to the high sugar levels in the samples. Therefore, 3SH and 3SHA were also analyzed using the ETP-SPE extraction method. However, neither thiol could be detected in any sample using either method. Both methods are shown to be efficient for free thiol quantification in a range of alcoholic beverages (Herbst-Johnstone et al. 2013, Tonidandel et al. 2021, Jelley et al. 2022), although there is insufficient report of their application in unfermented or lightly fermented “juice”-like media.

It is therefore of interest to investigate the effect of H_2_S production by yeast on early thiol output in real grape juice. In addition to the considerable inter-batch variabilities of nutrients, oxygen levels, and precursors, grape juice contains natural yeast and non-yeast microbial flora. Although chemical measures can be taken to suppress the growth of some unwanted microorganisms, some natural yeasts are selected for high tolerance of common antimicrobial treatments (e.g. SO_2_). This may confound the deduction of the H_2_S-thiol relationship using a pure starter culture with exogenous (*E*)-hex-2-enal precursor, since it will be rapidly metabolized by yeast and other microorganisms.

Moreover, an alternative pathway may compete with H_2_S for the precursor (*E*)-hex-2-enal in juice and GSH-containing SGM, which may also explain the lack of detectable early 3SH and 3SHA. It is hypothesized that the majority of available (*E*)-hex-2-enal could first conjugate with GSH by glutathione S-transferase, followed by conversion to cysteinylated precursor catalyzed by γ-glutamyl transferase and carboxypeptidase, before further downstream conversion by β-lysis to form 3SH (Kobayashi et al. 2011, Helwi et al. 2016, Thibon et al. 2016). Alternatively, (*E*)-hex-2-enal can conjugate directly with cysteine to form *S*-cysteine conjugates (Tominaga et al. 1998, Tominaga and Dubourdieu 2000, Starkenmann 2003, Starkenmann et al. 2008). It leads, however, to the question of the actual impact of H_2_S directly on final thiol production, either in the early stage or over the full course fermentation, as a major fraction of its target (*E*)-hex-2-enal would be sequestered by a plethora of nucleophilic attackers, including GSH and cysteine. This suggests that alternative pathways are likely to predominate since it is estimated that the contribution from direct formation of 3SH/3SHA may be limited to only ∼5% of final thiol output (Subileau et al. 2008, Harsch et al. 2013). Nonetheless, the lack of thiol detection in 12 h early fermentations alone could not exclude the possibility that the insufficient H_2_S accumulation in a laboratory setting led to poor thiol formation, while natural yeast populations may produce more sufficient H_2_S in response to nutritional stress (e.g. nitrogen limitation) (Henschke and Jiranek 1991, Jiranek et al. 1995, Bell and Henschke 2005).

### C_6_ precursor consumption during early fermentation

**Figure 3.**
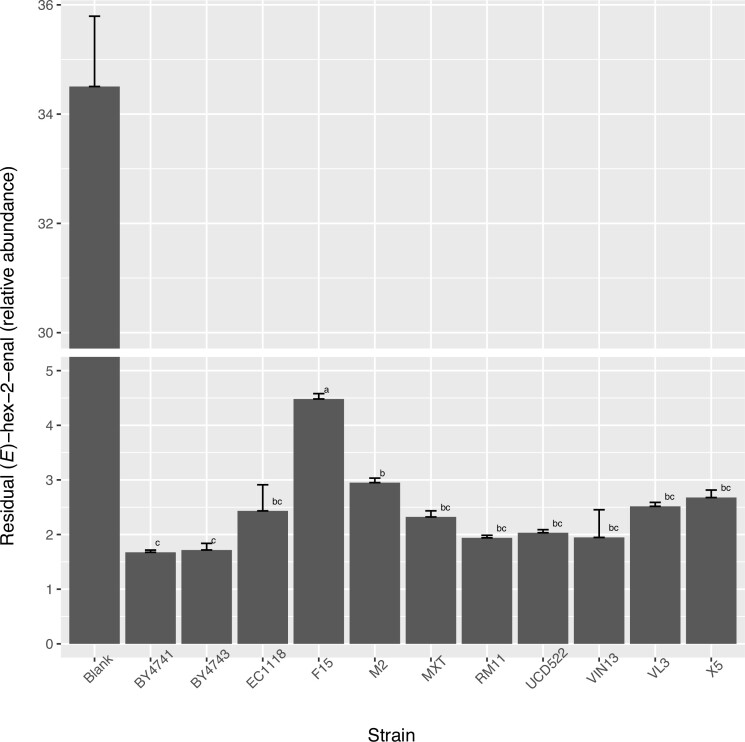
Post-fermentation residual level of (*E*)-hex-2-enal in fermentations. Data are presented as mean ± SEM. Means followed by a common letter are not significantly different by multiple comparison in post-hoc Tukey’s HSD test at 0.05 level of significance.

**Figure 4.**
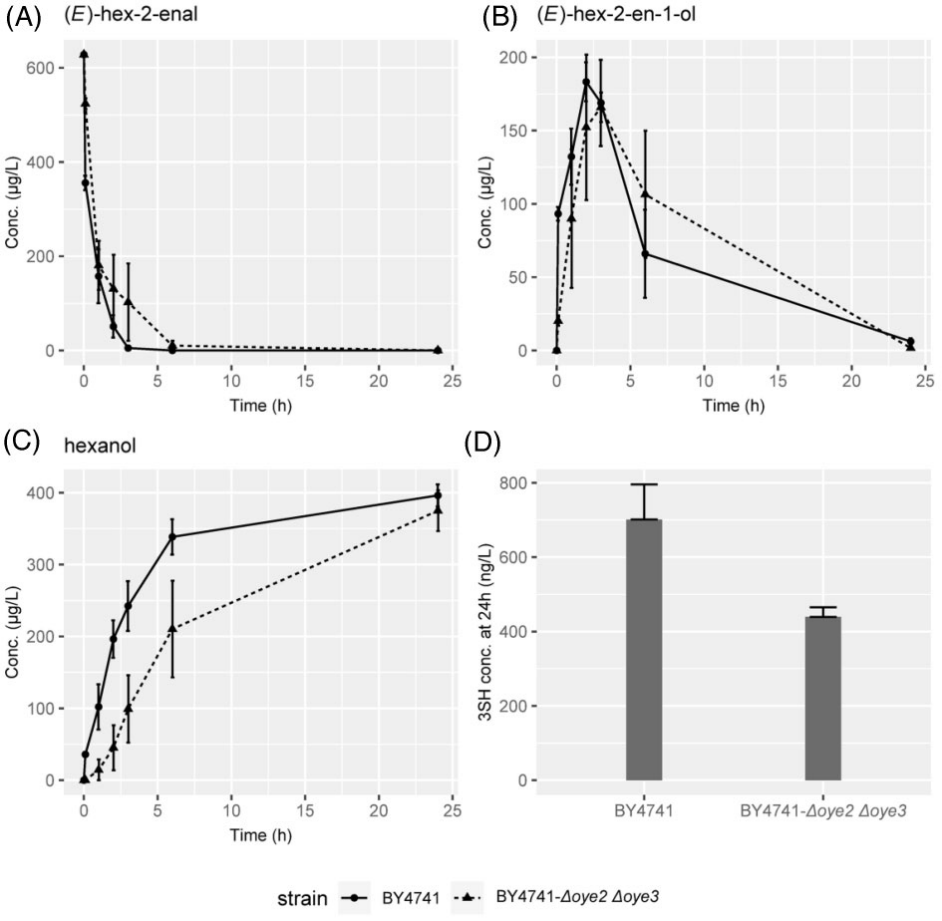
Early kinetics of C6 precursor and derivative compounds in the presence of BY4741 and BY4741-*Δoye2 Δoye3* double deletant in YNB medium. Levels of (*E*)-hex-2-enal (A), (*E*)-hex-2-en-1-ol (B), and hexanol (C) were monitored in the medium, at immediate (brief yeast exposure) (both 0 h), 1, 2, 3, 6, and 24 h. Spot thiol quantification carried out for 24 h samples (D), 3SHA was not detected in either group. Error bars represent mean ± SEM, *n* = 3.

The consumption of unsaturated C_6_ compounds, including (*E*)-hex-2-enal and (*E*)-hex-2-en-1-ol, was investigated. Consistent with previous reports, the concentration of C_6_ compounds significantly decreased in the presence of yeast regardless of strain tested (Fig. 3). An ANOVA showed statistically significant variabilities in the residual concentrations of C_6_ compounds among the 11 strains, which may suggest differences in abilities to withstand α,β-unsaturated aldehyde toxicity (Kubo et al. 2003, Matsui 2006, Ma et al. 2019). High residual concentrations of C_6_ compounds were detected in the fermentations produced from F15, a commonly used commercial wine strain with high thiol output; M2 also showed retarded early-stage C_6_ detoxification compared with laboratory strains BY4743 or BY4741. Moreover, a time-course assay was performed to track (*E*)-hex-2-enal concentration and its derivatives in the presence of BY4741 or BY4741-*Δoye2 Δoye3* double deletant. In both cases, the concentration of (*E*)-hex-2-enal decreased almost immediately after yeast inoculation and fell below the detection threshold within 10 h. Meanwhile, derivatives of (*E*)-hex-2-enal reduction accumulated in the fermentations (Fig. 4). Deletion of *S. cerevisiae* old yellow enzymes (*OYE*) genes *OYE2* and *OYE3*, which have been shown to be responsible for the reduction of α,β-unsaturated aldehydes (Williams et al. 2002, Trotter et al. 2006, Stuermer et al. 2007), had an insignificant impact on the elimination of (*E*)-hex-2-enal, suggesting possible pathway redundancy. Unsaturated aldehydes pose significant cellular toxicity and are therefore considered to be an antimicrobial mechanism against unwanted microorganisms. In turn, winemaking yeasts are selected for unsaturated C_6_-resistance, particularly due to the high unsaturated C_6_, or “green leaf aldehydes” released from damaged grape tissue during harvest and mechanical pressing (Joslin et al. 1978, Hammerbacher et al. 2019).

Early kinetics of C_6_ precursor consumption was also investigated in BY4741 and the BY4741-*Δoye2 Δoye3* double deletant, with the exogenous addition of (*E*)-hex-2-enal and H_2_S in the form of NaSH to ensure that 3SH would be produced (Fig. 4). C_6_ concentration diminished rapidly in the presence of yeast cell inoculation. In fact, even brief contact of yeast inoculant with (*E*)-hex-2-enal-spiked YNB medium, followed by yeast removal via centrifugation, was sufficient to almost halve the quantifiable (*E*)-hex-2-enal (second data point in Fig. 4A), which was consistent with the observation of rapid (*E*)-hex-2-enal elimination in previous reports (Joslin et al. 1978, Harsch et al. 2013). In fact, the precursor elimination occurred faster than previous estimations, as only trace levels of (*E*)-hex-2-enal were quantified after 5–6 h. Together with the non-detection of thiol compounds in 12 h ferments, it therefore raises the question of the direct contribution of H_2_S-C_6_ pathway, toward total thiol output in finished wine (Schneider et al. 2006, Subileau et al. 2008). Interestingly, in the presence of an exogenous source of H_2_S, we were able to detect 3SH at 24 h both with BY4741 (701.2 ± 94.6 ng L^−1^, mean ± SEM) and BY4741-*Δoye2 Δoye3* (439.27 ± 25.7 ng L^−1^, mean ± SEM) (Fig. 4D). 3SHA was not detected in either group. Although the *Δoye2 Δoye3* double deletion appeared to show reduced 3SH production after 24 h, there was no statistically significant effect observed (*P* = 0.0557); however, since this *P*-value was very close to the threshold set for significance (0.05), further investigation on the role of the *OYE* genes is warranted. It should be noted that the profile of H_2_S release from exogenous NaSH, which produces an initial burst of H_2_S within a relatively short time window, is drastically different from the slower and persistent endogenous H_2_S production from the yeast population (Harsch et al. 2013, Song et al. 2014). Further investigation using slow-releasing H_2_S donors may better mimic the accumulation and release of yeast endogenous H_2_S and its impact in the context of physiologically comparable kinetics (Kashfi and Olson 2013, Song et al. 2014).

*OYE* genes have been suggested to catalyze the reduction of α,β-unsaturated aldehydes (Williams and Bruce 2002, Yuan et al. 2011). Yet, deletion of *OYE2* and *OYE3* did not significantly impact the capability of BY4741 strain to metabolize (*E*)-hex-2-enal, suggesting redundancies or diversions in the detoxification pathway. Indeed, due to the large aldehyde reductase family in the *S. cerevisiae* genome, it is difficult to pinpoint the gene solely responsible for precursor metabolism.

## Conclusion

The dual role of H_2_S in VSCs and thiol compound production during the early stage of fermentation was investigated. To our knowledge, this preliminary work addresses, for the first time, the fine landscape of sulfur volatile compounds in the transitional stage of early fermentation. The variability of early H_2_S potential among yeast strains was found to have a significant influence on the chemical profile of VSCs. In light of the non-detection of 3SH/3SHA and rapid elimination of free C_6_ precursors in early ferments, our data does not support the direct contribution of endogenous H_2_S to early thiol output via the addition to the (*E*)-hex-2-enal precursor in SGM. However, upon addition of exogenous H_2_S, the elevated concentration of labile H_2_S results in the production of 3SH and 3SHA, suggesting that certain conditions must be met to reach sufficient H_2_S concentrations to obtain thiols from the (*E*)-hex-2-enal pathway. The development of efficient thiol quantification methods for the specific analysis of juice or juice-like substrates may assist in shedding further light on the early kinetics of thiol compounds during alcoholic fermentation.
